# Four-Dimensional Mesoscale Liquid Model of Nucleus Resolves Chromatin’s Radial Organization

**DOI:** 10.1103/PRXLife.2.013006

**Published:** 2024-01-30

**Authors:** Rabia Laghmach, Michele Di Pierro, Davit A. Potoyan

**Affiliations:** Department of Chemistry, Iowa State University, Ames, Iowa 50011, USA; Department of Physics, Northeastern University, Boston, Massachusetts 02115, USA; Department of Chemistry, Iowa State University, Ames, Iowa 50011, USA and Department of Biochemistry, Biophysics and Molecular Biology, Iowa State University, Ames, Iowa 50011, USA

## Abstract

Recent advances chromatin capture, imaging techniques, and polymer modeling have dramatically enhanced quantitative understanding of chromosomal folding. However, the dynamism inherent in genome architectures due to physical and biochemical forces and their impact on nuclear architecture and cellular functions remains elusive. While imaging of chromatin in four dimensions is becoming more common, there is a conspicuous lack of physics-based computational tools appropriate for revealing the forces that shape nuclear architecture and dynamics. To this end, we have developed a multiphase liquid model of the nucleus, which can resolve chromosomal territories, compartments, and nuclear lamina using a physics-based and data-informed free-energy function. The model enables rapid hypothesis-driven prototyping of nuclear dynamics in four dimensions, thereby facilitating comparison with whole nucleus imaging experiments. As an application, we model the *Drosophila* nucleus and map phase diagram of various possible nuclear morphologies. We shed light on the interplay of adhesive and cohesive interactions which give rise to distinct radial organization seen in conventional, inverted, and senescent nuclear architectures. The results also show the highly dynamic nature of the radial organization, the disruption of which leads to significant variability in domain coarsening dynamics and consequently variability of chromatin architecture. The model also highlights the impact of oblate nuclear geometry and heterochromatin-subtype interactions on the global chromatin architecture and local asymmetry of chromatin compartments.

## I. INTRODUCTION

Advances in genomics, computer simulations, and high-resolution microscopy have provided unprecedented insights into chromatin folding and its functional implications [1,2]. Three distinct mechanisms responsible for shaping nuclear architectures have emerged [3]: loop extrusion, phase separation, and chromatin anchoring to the nuclear envelope. The physical interactions driving these mechanisms are lengthwise compaction, chromatin locus cohesion, and chromatin locus adhesion to the nuclear membrane. The lengthwise compaction [4-7] originates from an interplay of equilibrium protein binding and nonequilibrium machinery that loop distal regions of chromosomes into topologically associating domains. Cohesive interactions [8,9], which lead to phase separation of chromosome regions, originate from the presence of epigenetically distinct A/B chromatin loci, which drive the formation of heterochromatin (HC) and euchromatin (EC) domains, respectively. Heterochromatin can be broadly classified into constitutive (cHC) and facultative heterochromatin (fHC) types, which have distinct structural, dynamical, and functional properties [10-12]. The origin of adhesive interactions between chromatin and the nuclear membrane [13] is mediated by filaments, collectively known as nuclear lamins. Lamins interact, directly or indirectly, with heterochromatin, resulting in preferential anchoring of heterochromatin to the nuclear membrane. Genomic regions that can anchor to the nuclear envelope can be identified in experiments and are referred to as lamina-associated domains (LADs) [14,15].

Chromatin architecture lives in an inherently active and stochastic environment surrounded by numerous proteins and RNAs, which constantly remodel and reorganize chromatin architecture [16-18]. At the same time, architectural dynamical patterns of chromatin organization are not random but instead are tightly coupled to the embryonic developmental timetable and cell cycle. Numerous experiments report on the radial organization of the genome’s physical features, including GC content gradients, transcriptional activity, and heterochromatin distribution relative to nuclear origin [19-23]. For instance, in healthy cells, nuclei adopt a conventional architecture with a distinct radial preference of heterochromatin regions towards nuclear envelope [24]. Conversely, heterochromatin gradually shifts toward the center in the senescent cells, often forming a few larger clusters [25]. During differentiation, heterochromatin may detach from lamina altogether, forming the so-called inverted nuclei, which are common in the rods of nocturnal mammals [24]. Cancer progression is likewise accompanied by a massive reorganization of heterochromatin regions near lamina [25,26]. Several fundamental questions remain unanswered regarding the role of lamina loss and its impact on chromatin organization during senescence and various diseases. The increasing sophistication in live nucleus imaging, 3D microscopy, and spatial transcriptomics [27-29] is now generating data that could benefit from mechanistic 3D nucleus modeling approaches. These advances make it possible to carry out mechanistic modeling of nuclei on mesoscopic scales, enabling the understanding of the mechanobiology of the nucleus.

To this end, we have developed a model of the nucleus termed the mesoscale liquid model of nuclear dynamics in four dimensions (MELON-4D). The model uses a physics-based and data-informed free-energy function to describe the dynamic evolution of chromosomal territories (CTs), compartments, and nuclear lamina. The physics-based part of the free-energy function is motivated by experimental features of the eukaryotic genome learned from Hi-C and imaging experiments. Specifically, the model accounts for the three fundamental driving forces of the chromosomal organization by explicitly modeling cohesive interactions between liquid chromatin phases, adhesive interaction with the lamina, and differential mobilities of euchromatin and heterochromatin due to different degrees of local compaction. At the same time, the model is not fitted to data. This has allowed us to study in detail the interplay of forces underlying key characteristics of three-dimensional (3D) nuclear architectures, including radial distribution of heterochromatin, domain-domain distances, shapes, and volumes of domains. Finally, we note that our results regarding the radial organization and its role as a gatekeeper of architectural variability are in harmony with the findings of recent computational models of *Drosophila* genome [19].

## II. MELON-4D

Here we explain MELON-4D, a model for describing the mesoscale motions of chromatin domains in 3D nuclear geometries. This model builds upon our previous phase-field approach, which focused on chromatin-type patterning in a fixed 2D geometry [30,31]. The previous model successfully recapitulated several experimentally observed phenomena, including phase-separation-driven interchromosomal coherent motions, chromatin patterning in aging and normal nuclei, and activity-enhanced droplet fusion dynamics. However, the 2D model of the nucleus had severe shortcomings such as the surface tension and fusion dynamics of chromatin compartments in 3D space. Below we summarize the multi-phase field formulation used in the MELON-4D model by highlighting key improvements relative to our previous approach. In MELON-4D, sets of phase-field variables $\varphi (r,t)=\{{\left\{\varphi_{i}(r,t)\right\}}_{i=1,\ldots ,N}\}$ and $\psi (r,t)=\{{\left\{\psi_{j}(r,t)\right\}}_{j=1,3}\}$ are introduced as nonconserved order parameters to describe the shape variation of various compartments within the nucleus that correspond to N-chromosomal territories and three types of chromatic regions of chromosomes which are in EC and fHC or cHC forms. Note that there are only two independent states of chromatin in the model described by $\psi_{1}$ and $\psi_{2}$ due to the constraint on the phase-field variables $\psi_{j}$ given by $\sum_{j=1}^{3}\psi_{j}(r,t)=1$. The phase-field variables $\varphi_{i}(r,t)$ and $\psi_{j}(r,t)$ vary smoothly across their interface profile between two values, 1 inside its compartment and 0 elsewhere.

The shape of an idealized eukaryotic nucleus is usually identified as ellipsoidal or spherical with a diameter ranging from 5 to 20 μm [32,33]. The geometry of the nucleus is defined through an auxiliary order parameter η(r,t), which takes the value 0 inside the nucleus and 1 outside and varies smoothly between these two values through the interfacial region. This region represents the nuclear envelope whose position is given by the isocontour $\eta (r,t)=\frac{1}{2}$. The phase-field variable η is used here as an indicator function independent of time to model a fixed nucleus with volume $V_{N}$. The nuclear envelope is assumed at the equilibrium state during the interphase of the cell cycle, which can be represented by a tanh-like profile of η(r). We simulated the oblate nuclear shape typical of eukaryotic nuclei during the interphase by the use of the expression $\eta (r)=\frac{1}{2}[1-\tanh(r∕2\sqrt{2}\epsilon_{\eta })]$, where $r=\sqrt{{\left(x∕a\right)}^{2}+{\left(y∕b\right)}^{2}+{\left(z∕c\right)}^{2}}$ is the distance from the center of the physical compartment Ω and a, b, and c are the semiaxes. The width of the nuclear envelope is given by $2\sqrt{2}\epsilon_{\eta }$.

The dynamics of the chromatin compartmentalization patterns are derived from the free-energy functional ℱ[φ,ψ], which describes the intranuclear phase separation of chromatin subtypes. The evolution of the phase-field variables {φ,ψ} are governed by the Allen-Cahn equations

(1) $$\frac{\partial \varphi_{i}}{\partial t}=-L_{\varphi }\frac{\delta ℱ[\varphi ,\psi ]}{\delta \varphi_{i}},\;\;i=1,\ldots ,N,\;\frac{\partial \psi_{1}}{\partial t}=-L_{\psi_{1}}\frac{\delta ℱ[\varphi ,\psi ]}{\delta \psi_{1}}+\zeta_{\psi_{1}}(r,t),\;\frac{\partial \psi_{2}}{\partial t}=-L_{\psi_{2}}\frac{\delta ℱ[\varphi ,\psi ]}{\delta \psi_{2}}+\zeta_{\psi_{2}}(r,t),$$

where $L_{\varphi }$, $L_{\psi_{1}}$, and $L_{\psi_{2}}$ are mobility coefficients that are proportional to the relaxation time of different phase-field variables. We have chosen the mobility coefficients to match the magnitudes of *in vivo* measurements of euchromatin and heterochromatin diffusion coefficients [34]. The terms $\zeta_{\psi_{j}}\;(j=1,2)$ in Eq. (1) account for the fluctuations at the boundaries of EC-fHC and EC-cHC islands due to the finite-size nature of the droplets. The fluctuations are modeled as white noise $\langle \zeta_{\psi_{j}}(r,t)\zeta_{\psi_{j}}(r^{\prime },t^{\prime })\rangle =A_{\psi_{j}}\delta (r-r^{\prime })\delta (t-t^{\prime })$, where the amplitude of the noise is given by $A_{\psi_{j}}={2k}_{B}TL_{\psi_{j}}$. The amplitude of noise $A_{p}$ sets the effective temperature $T_{\text{eff}}$ of the nucleus [35], which can be taken as a measure of ATP activity in comparison with the experiment [36,37].

The phase-field description of the nucleus in the present work is best described in terms of experimentally motivated constraints on shapes and sizes of the nucleus and chromosomal territories (CTs) [38], which are supplemented by the physics-based terms accounting for polymer intermingling diffusive segment motion within and between chromosomal territories. The primary driving forces for emergent nuclear architecture and dynamics are derived from the microphase separation of heterochromatin subtypes, the surface tension of chromatin droplets, and differential affinity for chromatin-lamina interactions. The chromatin types’ volume and surface constraints are imposed to capture chromosomal and nuclear boundaries. The full free-energy functional of the nuclear chromatin $ℱ\left[\varphi ,\psi \right]∕k_{b}T$, which we minimize in Eq. (1) to get the evolution equations of the nuclear structures, can be split into two energy contributions $F_{B}$ and $F_{I}$. The Ginzburg-Landau free-energy functional $F_{B}$ describes the coexistence of two phases associated with each phase-field variable completed by volume constraint terms to ensure the shape change given by

(2) $$F_{B}[\varphi ,\psi ]\;\;=\int_{\Omega }d\Omega \left[\sum_{i=1}^{N}\frac{\epsilon_{\varphi }^{2}}{2}{\left(\nabla \varphi_{i}\right)}^{2}+\sum_{j=1}^{2}\frac{\epsilon_{\psi }^{2}}{2}{\left(\nabla \varphi_{j}\right)}^{2}+f(\varphi ,\psi )\right]\;\;+a_{1}{\left[V_{N}-\sum_{i=1}^{N}V_{i}(t)\right]}^{2}+a_{2}\sum_{i=1}^{N}{\left[V_{i}(t)-{\bar{V}}_{i}(t)\right]}^{2}\;\;+a_{3}\sum_{i=1}^{N}{\left[v_{i}(t)-{\bar{v}}_{i}(t)\right]}^{2}+a_{4}\sum_{i=1}^{N}{\left[w_{i}(t)-{\bar{w}}_{i}(t)\right]}^{2},$$

where f(φ,ψ) is the bulk free-energy contribution for multiphase-field variables and gradients term accounting for the presence of different interfaces in the system and contributing to the interfacial energies. The gradient parameters $\epsilon_{\varphi }$ and $\epsilon_{\psi }$ control the thickness of the interface profile of φ and ψ, respectively. The standard form of the double-well potential for each phase-field variable (φ,ψ) is used here to describe the two coexistence bulk phases given by values 1 (inside the domain) and 0 (outside the domain). For the bulk free-energy density, we use thus a multi-well potential expressed as $f(\varphi ,\psi )=\sum_{i=1}^{N}\varphi_{i}^{2}{\left(1-\varphi_{1}\right)}^{2}∕4+\sum_{i=1}^{2}\psi_{i}^{2}{\left(1-\psi_{i}\right)}^{2}∕4$. Terms proportional to $a_{i}$ account for volume constraints required to enforce the volume of the chromosomal territories at their prescribed values ${\bar{V}}_{i}$, the facultative heterochromatin at ${\bar{v}}_{i}$, and the constitutive heterochromatin at ${\bar{w}}_{i}$. The parameters $a_{1}$, $a_{2}$, $a_{3}$, and $a_{4}$ are positive coefficients that control the thermodynamic driving forces of coarsening processes of different compartments present in the nucleus. The volumes of the i-chromosomal territory $V_{i}(t)$ and facultative and constitutive heterochromatin compartments within each chromosome $v_{i}(t)$ and $w_{i}(t)$ are defined as the spatial integral over the physical compartment Ω of their interface profiles given by the associated phase-field variables $\varphi_{i}(r,t)$, $\psi_{1}(r,t)$, and $\psi_{2}(r,t)$. Using the usual phase-field approximation of the volume could change the coexistence phase values defined by the phase-field variables between 0 and 1. We thus used an interpolation function h for approximating the volumes of different compartments of the nucleus while keeping the position of the local free-energy minima at the coexistence phase values. The most frequently adopted polynomial function for calculations of the bulk free-energy density using diffuse interface methods [39] is $h(\varphi_{i})=\varphi_{i}^{3}(10-15\varphi_{i}+6\varphi_{i}^{2})$. The following expressions approximate the volumes of these compartments within the nucleus: $V_{i}(t)=\int_{\Omega }d\Omega \;h(\varphi_{i})$, $v_{i}(t)=\int_{\Omega }d\Omega \;h(\psi_{1})h(\varphi_{i})$, and $w_{i}(t)=\int_{\Omega }d\Omega \;h(\psi_{2})h(\varphi_{i})$.

Next we define the free-energy functional $F_{I}$, which accounts for the geometrical constraints on the nucleus, excluded-volume interactions between chromosome territories and different chromatin compartments within the nucleus, and adhesive interactions between heterochromatic subtypes with the nuclear envelope. The functional $F_{I}$ is expressed as

(3) $$F_{I}[\varphi ,\psi ]=\beta_{0}\sum_{i=1}^{N}\int_{\Omega }d\Omega \;h(\eta )[1-h(\eta )]h(\varphi_{i})\;\;+\beta_{\varphi }\sum_{i\neq j}^{N}\int_{\Omega }d\Omega \;h(\varphi_{i})h(\varphi_{j})\;\;+\beta_{\psi_{1},\psi_{2}}\int_{\Omega }d\Omega \;h(\psi_{1})h(\psi_{2})\;\;+\int_{\Omega }d\Omega \left[1-\sum_{i=1}^{N}h(\varphi_{i})\right][\beta_{\psi_{1}}h(\psi_{1})+\beta_{\psi_{2}}h(\psi_{2})]\;\;+\int_{\Omega }d\Omega \;g(\nabla \eta ,\nabla \psi ).$$

The first term in $F_{I}$ corresponds to the energy penalty reflecting the geometrical constraint on the nuclear volume required to restrain nuclear components’ motion inside the nucleus. The $F_{I}$ works by increasing the surface tension of the nucleus when chromosomal territories are near the nuclear envelope. The other terms represent the excluded-volume interactions between chromosome territories (CTs), fHC-cHC region interactions, and HC-EC region interactions, where the interactions strengths are controlled by the positive nondimensionalized parameters $\beta_{\varphi }$, $\beta_{\psi_{1},\psi_{2}}$, $\beta_{\psi_{1}}$, and $\beta_{\psi_{2}}$. The last term describes the effect of nuclear lamina-heterochromatin tethering through a g function, which represents the local lamin-heterochromatin adhesion energy

$$g(\nabla \eta ,\nabla \psi )≕\nabla h(\eta )\cdot [\gamma_{1}\nabla h(\psi_{1})+\gamma_{2}\nabla h(\psi_{2})],$$

where $\gamma_{1}$ and $\gamma_{2}$ are two positive parameters controlling the binding affinity of heterochromatin types to the nuclear lamina. The values $\gamma_{1}=\gamma_{2}=0$ indicate no adhesion between heterochromatin types and nuclear lamina. Increasing the value of $\gamma_{i}$ leads to strong nuclear lamina adhesion of heterochromatin i type.

### Parametrization of MELON-4D

By introducing a characteristic length of spatial resolution l and time τ, the phase-field equations of chromatin dynamics are written in their dimensionless forms. To numerically solve the set of evolution equations of the phase-field variables resulting from the free-energy functional minimization (1), we use the finite-element method combined with the preconditioned Jacobian-free Newton-Krylov approach. The model has been implemented in the moose finite-element c++ library [40,41], which is built using high-performing computational libraries mpi, libmesh, and petsc needed for solving nonlinear partial differential equations [41]. Once all the evolution equations of the phase-field variables are transformed in the weak form to extract the residual vectors, we compute the solution on a computational domain denoted by Ω and perform 3D simulations of chromatin dynamics within an ellipsoidal nucleus.

The parameters involved in the MELON-4D model are estimated from nucleus imaging experiment data. The estimated size of the *Drosophila* genome [42,43] is between 150 and 180 Mb with a nuclear diameter of around 5 μm. Nearly a third of the nuclear volume is occupied by heterochromatin. The model resolves chromatin dynamics at the approximate Mb scale of spatial resolution discriminating between chromatin compartments. The length scale that makes the evolution equations dimensionless is fixed at l=1 μm. The introduced characteristic relaxation time τ is equivalent to the inverse of interfaces mobility $\tau \propto L^{-1}$, which is used to set the timescale of the dynamics of chromatin phase separation by rescaling times in evolution equations (1). The mobilities of chromosomal and heterochromatin compartments are set to be equal $L=L_{\varphi_{i}}=L_{\psi_{i}}$ in all the simulations. It is noted here that the value of τ is naturally expected to differ for different developmental stages of the nucleus. By exploring experimentally measured diffusion coefficients, we estimate realistic values of τ for the postembryonic interphase of *Drosophila* [44] to 0.005 s, while for studying long-time senescence and nuclear inversion, the timescale could be calibrated to match different sets of experiments [45] and corresponds to 5 h. We hence set the value of the timescale such that τ=0.005 s in this work. The diffusion coefficient can be expressed as $D_{\varphi_{i}}={L\epsilon }_{\varphi_{1}}^{2}$ for chromosomal territories, $D_{\psi_{1}}={L\epsilon }_{\psi_{1}}^{2}$ for the heterochromatin, and $D_{\psi_{2}}={L\epsilon }_{\psi_{2}}^{2}$ for the chromocenter. For all the simulations, the diffusion coefficients are fixed as $D_{\varphi }=20$ μm^2^/s and $D_{\psi_{1}}=D_{\psi_{2}}=12$ μm^2^/s. These values of diffusion coefficients are motivated by experimental measurements of euchromatin and heterochromatin mobility in live cells [34].

The computational domain used here is set as $\Omega =[0,L_{x}]\times [0,L_{y}]\times [0,L_{z}]$, with $L_{x}=6$ μm, $L_{y}=9$ μm, and $L_{z}=3$ μm, and we meshed this domain to generate a fine mesh made up of 180×225×90 elements. The time step used for time integration is set to 0.04 in dimensionless units, which is chosen to ensure the numerical stability of all simulations. The initial conditions used here to generate an elliptical nucleus in the center of the computational domain and nuclear architectures described by phase-field variables are provided by a tanh-like function given by $1∕2[1-\tanh(r∕2\sqrt{2}\varepsilon_{.})]$, where r is the distance from the center of the computational domain. The dimension of the simulated nucleus is given by the semiaxis values that are fixed to a=2.5 μm, b=4 μm, and c=1.2 μm, with a nuclear volume of $V_{N}=\frac{4}{3}\pi abc=50.26$ μm^3^ consistent with empirical measurements of the *Drosophila* nucleus during the interphase [46]. The spatial coordinates of the eighth chromosomal territories (N=8) within the nucleus are given as $X_{i}=\{(3,\;1.2,\;1.5)$; (1.2, 2.9, 1.5); (2.7, 3.25, 0.26); (4.5, 2.5, 1.5); (3.2, 5.4, 1.5); (4.8, 6.0, 1.5); (3.0, 8.2, 1.5); (1.35, 6.1, 1.5)}, in which heterochromatin domains are generated in the center of each chromosomal territory. The volume fraction of heterochromatin is assumed here as 25% of the nuclear volume.

The dimensionless parameters controlling excluded-volume interactions were chosen to account for the effect of the restricted positioning of chromosomes within the interphase nucleus and restrict the overlap. We have chosen the interaction coefficient between chromosomal territories and the nuclear envelope such that $\beta_{0}=16.7$, which is strong enough to restrict the movement of chromosomes within the nucleus. To ensure well-separated chromosome territories, the value of the dimensionless parameter was chosen as $\beta_{\varphi }=40$ such that the chromosome-chromosome interaction is strong like the one in [30] and also motivated by the shown phase diagram of 3D nuclear morphologies, which will be discussed later. The heterochromatin-heterochromatin interaction parameter is taken as $\beta_{\psi_{1}}=0.1$ for all simulations to ensure attraction between heterochromatin regions and thereby mimic liquidlike fusion of heterochromatin droplets within the nucleus. The two dimensionless parameters controlling the interaction strength of the chromo-chromocenter $\beta_{\psi_{2}}$ and hetero-chromocenter $\beta_{\psi_{1},\psi_{2}}$ are varied to investigate their role in generating interesting nuclear chromatin morphologies. We performed simulations for different values of the dimensionless interaction parameter between heterochromatin and nuclear lamina $\gamma_{i}$ to evaluate the effects of competition between binding energies and chromatin compartment interactions. The remaining model’s dimensionless parameters are fixed to enable dynamics comparable to chromatin diffusion coefficients such that $a_{1}=0.16$ and $a_{2}=a_{3}=a_{4}=2$. The fluctuation amplitude of euchromatin-heterochromatin and euchromatin-chromocenter interfaces $A_{1}$ and $A_{2}$ are fixed at 5. While the nuclear shape, size, chromosome numbers, and diffusion coefficients are calibrated after the *Drosophila* nucleus, the model is sufficiently general to draw broader conclusions about the chromatin structure and dynamics in eukaryotic nuclei. Note that the dimensionless coefficients $\beta_{\psi_{1},\psi_{2}}$, $\beta_{\psi_{2}}$, and $\gamma_{2}$ in the free-energy functional are set equal to zero for the two-component chromatin model, and only the phase-field variable $\psi_{1}$ remains to describe the chromatin density within the nucleus locally. In the following section with two-component of chromatin we omit the index 1 such that $\psi_{1}\equiv \psi$, $\gamma_{1}\equiv \gamma$, and $\beta_{\psi_{1}}\equiv \beta_{\psi }$.

**FIG. 1.**
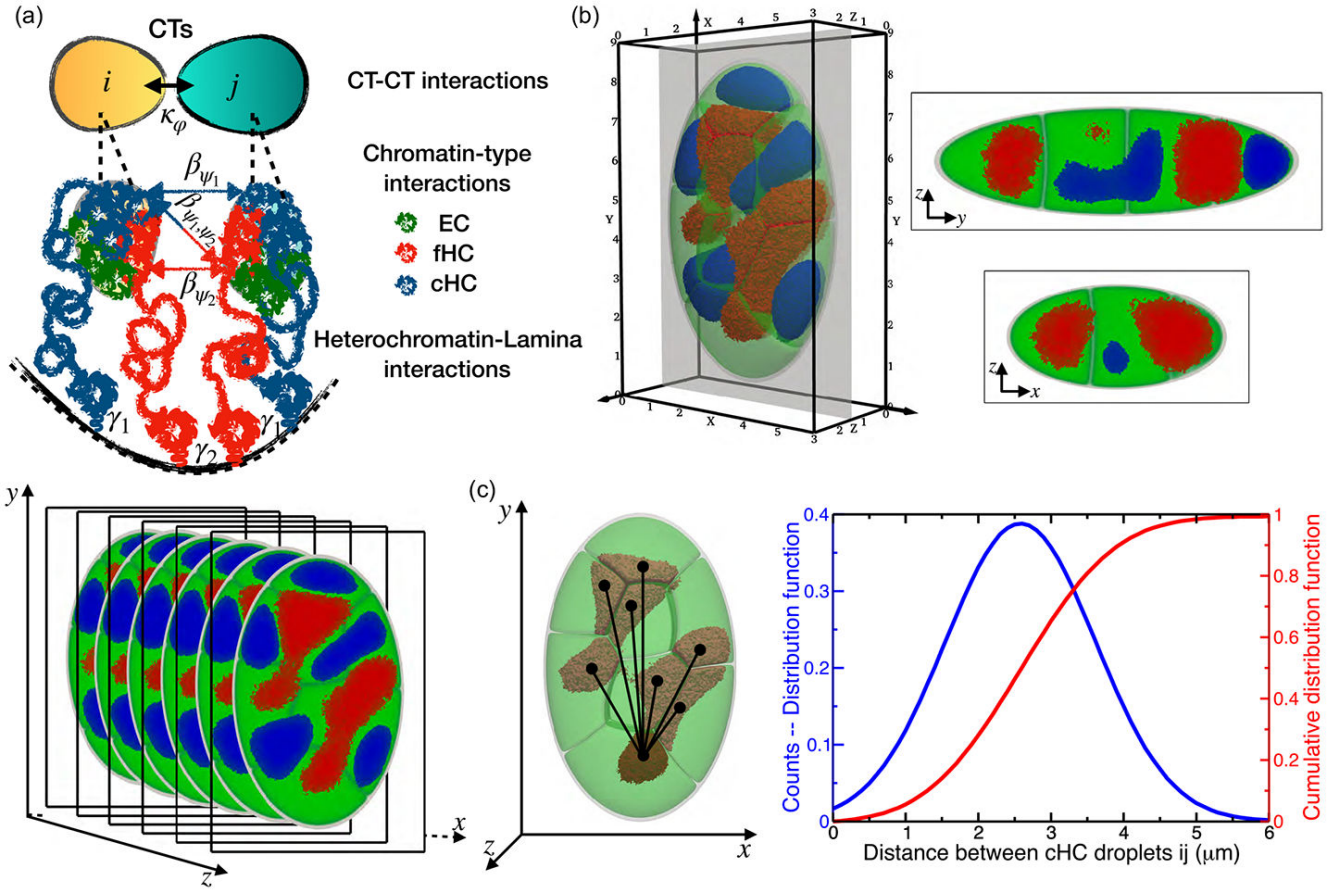
(a) 3D snapshots of a model *Drosophila* nucleus simulated using the MELON-4D framework which is resolving three chromatin types: euchromatin (green), facultative (red), and constitutive heterochromatin (blue). (b) 2D slice of simulated nuclear architecture along the x-y, y-z, and x-z axes. (c) Distribution of distances between the heterochromatin compartment centroids from the i-th and j-th chromosomes (j≠i).

To investigate the spatial characteristics of the resulting simulations of three-dimensional chromatin patterns, we have examined the distance of chromatin to the nuclear center and the cumulative distribution function of distances between heterochromatin droplets. We also analyzed the dimensions and shape of heterochromatin compartments from our 3D simulations by computing the volume distribution of the two types of HC droplets and the distribution function of the sphericity index given by [47] $\Psi =\pi^{1∕3}{\left(6V_{i}\right)}^{2∕3}∕S_{i}$, where $V_{i}$ and $S_{i}$ are the volume and surface of the object i. Figure 1 represents a schematic view of the MELON-4D framework resolving the dynamics of three chromatin states within the nucleus and summarizes the physical interactions between chromatin regions considered in the model.

## III. RESULTS

We model the *Drosophila* nucleus using an elliptical geometry containing eight chromosomal territories N=8. Each chromosome is resolved at the level of chromatin types corresponding to epigenetically distinct euchromatin and heterochromatin compartments. In the following two sections we look at the architecture and the dynamic evolution of heterochromatin compartments formed with one-component and two-component nuclei corresponding to cHC and fHC.

In MELON-4D, all phase-separated chromatin compartments are defined by phase-field variables tracking interfaces, volumes, and geometries of chromatin compartments. To evaluate how the interactions between chromatin types impact the organization and dynamics of compartments, we perform simulations of the 3D nucleus by varying the interaction strengths between chromosomal territories and different chromatin types. Note that the interactions between chromosomal territories govern the degree of intermingling between neighboring CTs and the global arrangement of chromosomes in the interphase nucleus. The CTs interactions are long ranged and correspond to the nucleus’s slowest timescale of chromatin motions. The CTs interactions are controlled by the parameter $\beta_{\varphi }$. Microphase separation and motion of chromatin compartments correspond to faster timescale motions in the nucleus. The parameter $\beta_{\psi_{1}}$ in the two-component chromatin model controls the attraction strength between EC and HC within individual CTs. Higher values of $\beta_{\psi_{1}}$ correspond to stronger attraction between chromatin types within CTs. Likewise, two parameters controlling attraction strengths between EC and HC subtypes are $\beta_{\psi_{1}}$ and $\beta_{\psi_{2}}$ corresponding to constitutive and facultative heterochromatin, respectively. The strength of interaction between the HC subtypes is given by $\beta_{\psi_{1},\psi_{2}}$. A higher value of $\beta_{\psi_{1},\psi_{2}}$ indicates weak attraction between the subtypes of heterochromatin. Thus, increasing the value of this parameter could lead to phase-separated heterochromatin compartments.

### A. Nuclear organization resolved with a two-component chromatin model

Here we consider two interacting chromatin types, euchromatin and heterochromatin. The dynamics and morphology of heterochromatin compartments in the long-time limit are controlled by chromatin-type interactions within chromosomes and the degree of intermingling between neighboring chromosome territories.

**FIG. 2.**
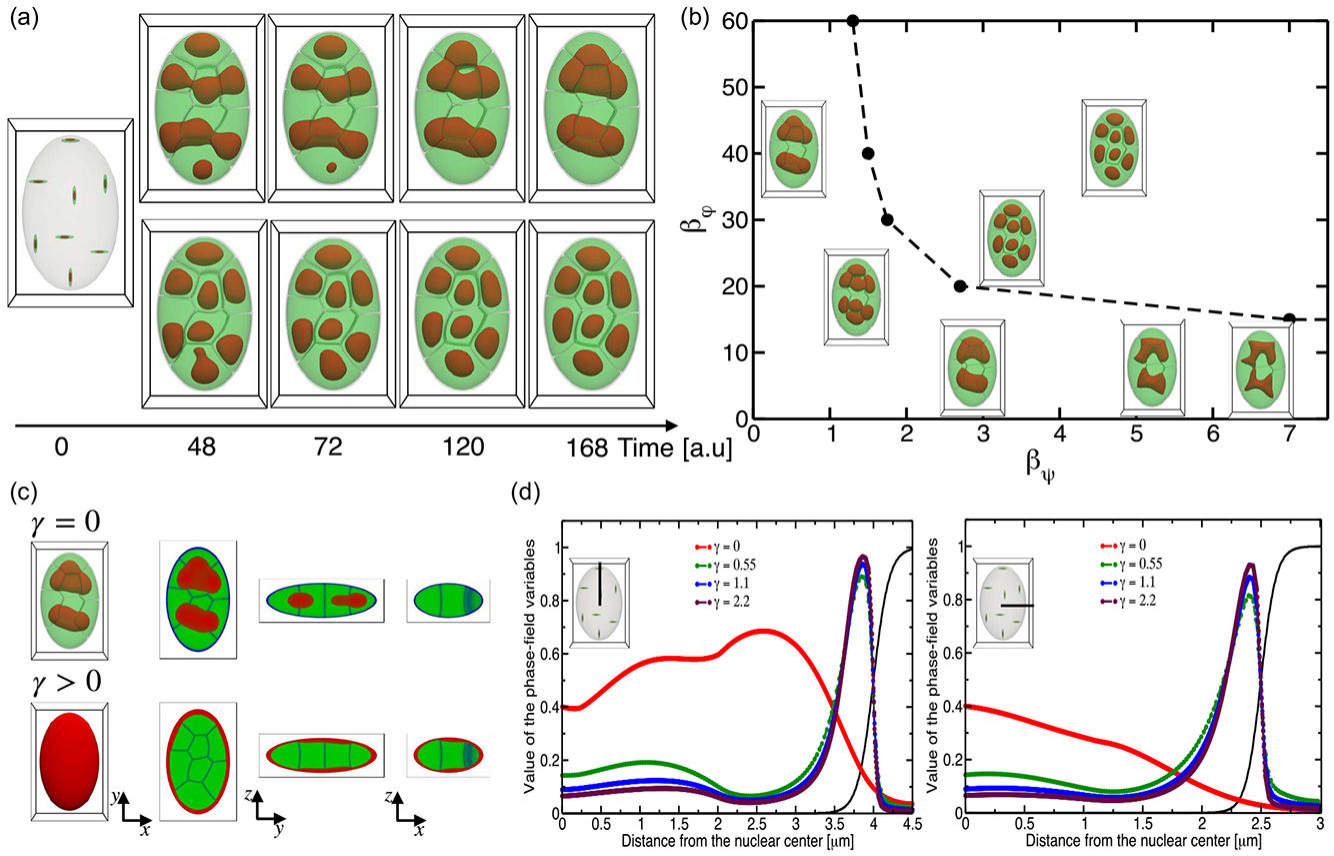
Temporal evolution of chromatin A/B patterns in three dimensions. (a) Effect of chromatin-type interactions on the degree of spatial compartmentalization of the nucleus. The simulations reported in (a) were performed with the fixed values of $\beta_{\varphi }=40$ and γ=0. The top panel shows the emergent chromatin pattern driven by a weaker attraction between chromatin types within chromosomes $\left(\beta_{\psi }=0.1\right)$ and the bottom panel shows stronger attraction $\left(\beta_{\psi }=4.5\right)$. (b) Phase diagram of nuclear chromatin architectures as a function of the two parameters controlling the strength of interactions between chromosomes and chromatin types $\beta_{\varphi }$ and $\beta_{\psi }$, respectively. (c) 3D nuclear architectures and 2D slices corresponding to the absence (γ=0) and presence (γ>0) of adhesive lamina-heterochromatin interactions in the top and bottom panels, respectively. (d) Radial density profile of heterochromatin along the major and minor axes computed from the nuclear center as a function of adhesive lamina-heterochromatin interaction strength. The simulations reported in (c) and (d) were performed with the fixed values of $\beta_{\varphi }=40$ and $\beta_{\psi }=0.1$.

First, we carried out 3D simulations with a fixed strength of CTs interaction $\left(\beta_{\varphi }=40\right)$, in the absence of nuclear lamina-HC anchoring (γ=0). Figure 2 shows the summary of diverse 3D patterns of A/B chromatin compartmentalization which emerge from competition between different types of CT-CT, A/B chromatin, and lamina-HC interactions. Predictably, for stronger chromatin type-to-type attraction, we find disconnected heterochromatin droplets in individual chromosomes [Fig. 2(a), bottom panel]. For weaker chromatin type-to-type attraction, we find connected heterochromatin droplets of different chromosomes residing in the interior of the nucleus [Fig. 2(a), top panel]. Furthermore, weaker chromatin type-to-type attraction revealed a more pronounced clustering of heterochromatin droplets, resulting in two large compartments localized within the interior of the nucleus [Fig. 2(a), top panel]. The nuclear structure obtained with stronger chromatin type-to-type attraction showed that heterochromatin droplets formed within chromosomes maintained their positions in the center of each CTs and are surrounded by euchromatin. Thus an increase in chromatin type-to-type attraction drives the localization of heterochromatin compartments within the chromosomal territory. Interestingly, in our previous work using a two-dimensional model of the nucleus [30], the nuclear structure that emerged with stronger chromatin type-to-type attraction exhibited fewer heterochromatin droplets clustering in the interior of the nucleus. This result shows the importance of 3D motions even for oblate geometry, which more accurately accounts for spatially interacting chromatin types that regulate the movement and clustering of the formed heterochromatin droplets.

The interplay between CTs and chromatin type-to-type interactions without considering the effect of nuclear lamina-HC interactions (γ=0) can span a wide spectrum of nuclear architectures. Therefore, we next vary only the two parameters controlling the strengths of physical interactions between CTs and chromatin types in the nucleus, $\beta_{\varphi }$ and $\beta_{\psi }$, respectively. The results of our 3D simulations are presented as phase diagrams of nuclear architectures showing the connectivity between heterochromatin droplets of different chromosomes [Fig. 2(b)]. We identify three distinct morphological regimes in the phase diagram. The first morphological regime corresponds to stronger chromatin type-to-type but weaker CTs interactions, driving disconnecting heterochromatin droplets formed within individual chromosomes. The second regime corresponds to weaker chromatin-type and CTs interactions, which drive the formation of strongly connected heterochromatin droplets localized near the center of the nucleus. The last regime corresponds to stronger chromatin type-to-type and CTs interactions, which drive the formation of strongly connecting heterochromatin droplets across the boundaries of chromosome territories. We note that nuclear morphologies generated by modeling have been observed in experiments on eukaryotic nuclei in stages of cell life where the connection with the lamina is severed, including embryonic growth of some species, inversion, and senescence [22,24,48,49].

Having considered cohesive interactions between chromatin types in 3D nuclei, we next turn to the type-specific adhesive interactions between chromatin and the lamina and analyze radial profiles and volumetric characteristics of heterochromatin-enriched domains. Radial profiles of *Drosophila* and other nuclei have been measured in recent whole genome experiments [22,42,50] and explored in the whole nuclei simulations of *Drosophila* [19], pointing out their role in generating robust nonrandom average global architectural patterns of heterochromatin, which is relatively insensitive to type interactions. Experimentally, it is known that heterochromatin compartments are partially tethered to the nuclear lamina for healthy *Drosophila* and other eukaryotic nuclei [19,50]. A preferential interaction between heterochromatin and the nuclear lamina could be sufficient to drive the motion of heterochromatin towards the nuclear periphery and lamina anchoring of heterochromatin droplets.

In a two-component chromatin model with sufficiently adhesive interactions γ>0 [Figs. 2(c) and 2(d)], we can capture nuclear architectures with radial profiles resembling the experiments and whole nucleus simulations of *Drosophila* [19,50]. Figures 2(c) and 2(d) show the results of 3D simulations for the fixed values $\beta_{\varphi }=40$ and $\beta_{\psi }=0.1$ and for different values of nuclear lamina-HC interaction strengths. By looking at the global features of radial profiles in 3D nuclei, we find the importance of analyzing both major and minor axes since some of the domain formations may be masked when taking a 2D slice representation of nuclei, which is done in experiments and 2D continuum models [30]. We found that the strength of the adhesive interaction, besides impacting chromatin 3D architecture, also has consequences on the dynamics of heterochromatin-droplet formation around the nuclear envelope. More specifically, stronger heterochromatin adhesive interaction generates rapid quenching of heterochromatin dynamics, leading to differences in 2D radial profiles between the major and minor axes [Fig. 2(d)]. Overall the results demonstrate how the interplay of chromatin interactions could regulate chromatin dynamics within the nucleus and shape conventional and inverted nuclear architectures. Additionally, the 2D slice-based representation shows that the chromatin distribution and variation in the size of heterochromatin compartments are spatially associated with the characteristic dimensions of the nucleus [Fig. 2(c)]. Based on this observation, we hypothesize that the differences in lamina attachment rates can impact gene expression variability, which can be quantified by studying the variability of heterochromatin layer thicknesses near the lamina.

### B. Nuclear organization resolved with a three-component chromatin model

Here we consider the three-component liquid chromatin model of the nucleus which can account for additional interactions taking place between chromatin subtypes, namely, constitutive cHC and facultative fHC types of heterochromatin. Physical interactions between CTs and all distinct pairs of chromatin subtypes (cHC, fHC, and EC) now govern the motion of heterochromatic compartments through the chromosomal boundaries, resulting in distinct architectures. Note that we did not account for the effect of nuclear lamina anchoring of heterochromatin types. Therefore, the parameters $\gamma_{1}$ and $\gamma_{2}$ are set to zero for all the remaining 3D simulations of the ternary chromatin-type systems.

**FIG. 3.**
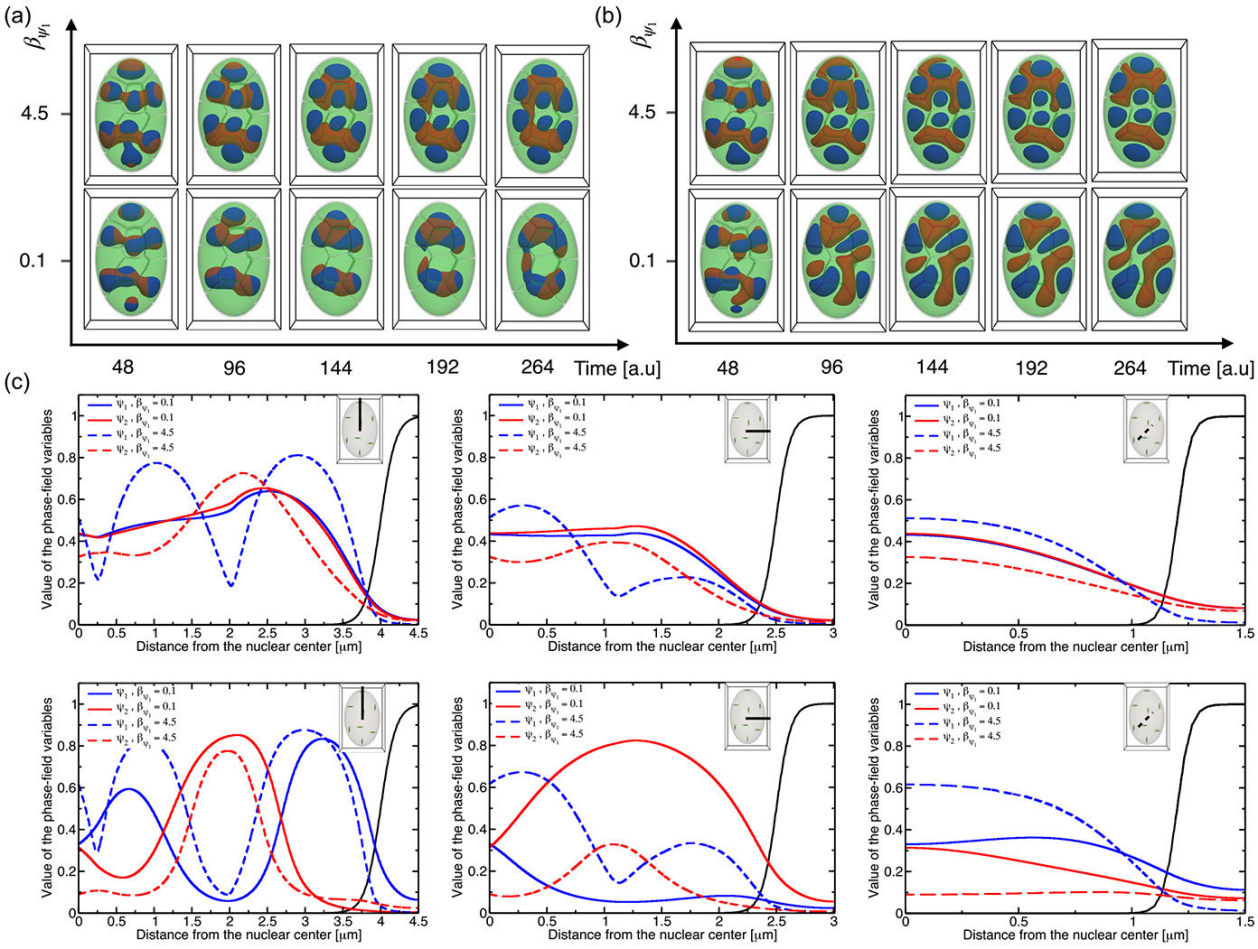
3D chromatin architectures of the nucleus resolved with three interacting chromatin types: EC (green), fHC (red), and cHC (blue). (a) Nuclear architectures resulting from simulations with constant strong, cohesive interactions between cHC and fHC with varying interaction strength between EC and cHC and between EC and fHC. (b) Nuclear architectures result from simulations with weak cohesive interactions between cHC and fHC with varying interaction strength between EC and cHC and between EC and fHC. (c) Radial density profiles of cHC and fHC along the major, minor, and z axes. The top panel corresponds to strong, cohesive interactions between cHC and fHC with varying interaction strength between EC and cHC and between EC and fHC. The bottom panel corresponds to weak cohesive interactions between cHC and fHC with varying interaction strength between EC and cHC and between EC and fHC.

To assess the sensitivity of the global radial order of the nucleus compartmentalization to chromatin-subtype interactions, we performed 3D simulations by varying the interaction strength of $\beta_{\psi_{1}}$ between cHC and EC and of $\beta_{\psi_{1},\psi_{2}}$ between cHC and fHC types of chromatin. Having shown that maintaining separated chromosomal territories with the possibility of overlapping within the intersections of their interfacial regions required setting weak interactions between chromosomes such that $\beta_{\varphi }=40$, we have chosen to set weak fHC-EC attraction $\left(\beta_{\psi_{2}}=0.1\right)$ to give more freedom for fHC droplets to move through CTs which favor fHC clustering at the interior of the nucleus. The values of $\beta_{\varphi }$ and $\beta_{\psi_{2}}$ are fixed in all simulations reported in this section. The results of our 3D simulations are presented in Fig. 3. Analysis of simulations shows the emergence of different nuclear morphologies governed by the degree of demixing of chromatin types and connectivity of heterochromatin droplets [Figs. 3(a) and 3(b)].

Phase-separated HC-droplet formation of varying demixed heterochromatin states depend on the strength of interactions between HC types: Increasing interaction strength $\beta_{\psi_{1},\psi_{2}}$ leads to demixing heterochromatin states and forming fully separated HC droplets. On the other hand, lowering the strength of interactions between HC and EC types within CTs leads to stronger cohesion of HC droplets from neighboring chromosomes that merge into large HC droplets. Simulations show that interactions between HC and EC types of chromatin within individual CTs in the lower-range values of $\beta_{\psi_{1}}$ and $\beta_{\psi_{2}}$ are required to establish a physical connection between similar types of HC droplets, while the distance between the different types of HC droplets increases with the strength of interactions between HC types. As shown, both types of heterochromatin droplets located within CTs display fast motion toward the chromosomal boundaries and clustering in larger HC compartments in the interior of the nucleus for the case $\beta_{\psi_{1}}=\beta_{\psi_{2}}=0.1$. In this case, we noticed a mixing of chromatin types close to the center of the nucleus driven by a strong adhesive interaction between the two HC types. For the case where the cHC-EC attraction is stronger than the fHC-EC attraction, i.e., $\beta_{\psi_{1}}=4.5$ and $\beta_{\psi_{2}}=0.1$, we observed a slow motion of cHC droplets that restricted their localization at the center of chromosomes, while fHC droplets moved fast toward the center of the nucleus and clustered in larger compartments. In the remaining cases, we observed demixed chromatin states where the two types of clustered HC are partially or fully disconnected. We also noticed that the number of fHC clusters within the nucleus is higher than the cHC clusters.

To quantify the effect of multicomponent interactions on the spatial organization of chromatin types in the nucleus, we computed the local density profiles of heterochromatin types along the nucleus’s major, minor, and z axes [Fig. 3(c)]. The density profiles of both heterochromatin types are similar in the case of strong cHC-fHC attraction compared to EC-cHC and EC-fHC interactions, which indicates a mixing state of heterochromatin types residing within the nuclear interior. When the cHC-EC attraction is stronger than the fHC-EC interaction, the profiles display demixing states of chromatin whose formed HC droplets are close to each other with the separation distance between them depending on the strength of cHC-fHC interactions. The density profiles show that, unlike fHC droplets, the cHC droplets reside closer to the nuclear center.

Interestingly, the distance between HC droplets is only slightly influenced by the strength of cHC-EC interactions. Results show that lowering the intensity of cHC-fHC attraction leads to maintaining separated HC droplets. As can be seen in Fig. 3, the merged fHC droplets are partially in contact with the centered cHC droplets within CTs for strong cHC-EC attraction, while they are fully separated for weak cHC-EC attraction.

To further quantify the larger-scale spatial arrangement of chromatin compartments, we follow the approach used in [51] based on evaluating spatial descriptors to compute the distance function between similar types of heterochromatin, volumes, and shapes of cHC and fHC compartments through sphericity values. We calculated the cumulative distribution function of the distance between the centroid of similar-type HC-droplet positions. We also evaluated the distribution of HC-droplet volumes and associated sphericity.

**FIG. 4.**
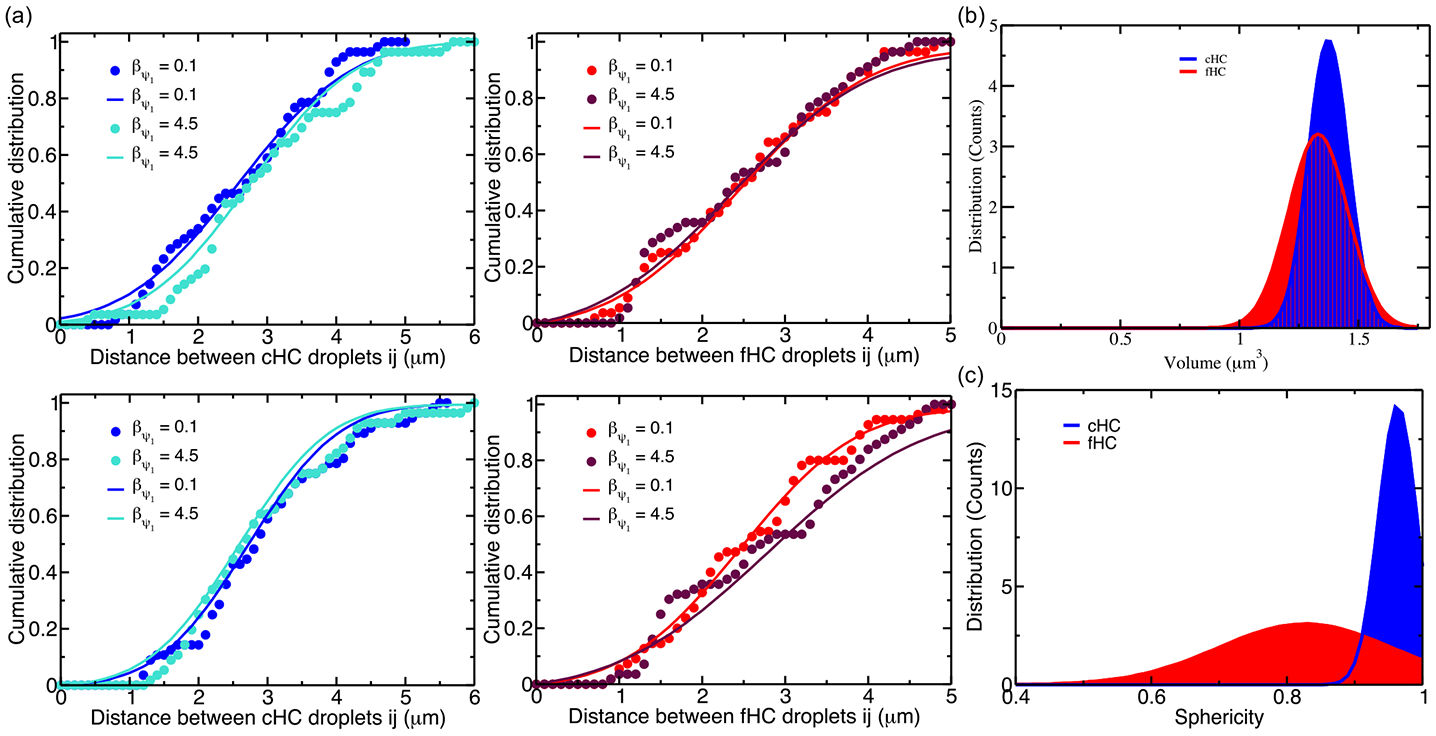
(a) Cumulative distribution of distances between the centroid of ith fHC droplets (ith cHC droplets) from the i chromosome and the centroids of all remaining jth fHC droplets (jth cHC droplets) from all j chromosomes (j≠i). The dashed line corresponds to the value obtained from the cumulative distribution of distance calculation and the solid line corresponds to the fitted value. (b) Distribution function of the volume of heterochromatin compartments. (c) Distribution function of the sphericity of heterochromatin compartments.

The cumulative distribution functions (Fig. 4) show longer distances between cHC droplets for simulations generated with the strong attraction between chromatin types cHC-fHC and EC-cHC. The case with stronger cHC-fHC attraction than EC-cHC attraction shows increased distances between cHC droplets. One can notice that the distance distribution between fHC droplets is independent of the strength of cHC-EC interactions for the simulated nucleus generated with strong cHC-fHC attraction. We find the inverse tendency for the distance distribution between HC droplets in the case with weak cHC-fHC attraction. In this case, we observed more considerable distances between fHC droplets for strong EC-cHC attraction.

The distributions of volumes of HC droplets are centered at 1.35 and 1.45 μm^3^ for fHC and cHC droplets, respectively, and it can be seen that the standard deviation for volume distribution of fHC droplets is more significant than that of cHC droplets [Fig. 4(b)]. We also noticed the same behavior for the distribution of HC-droplet sphericity [Fig. 4(c)]. The distribution of cHC-droplet sphericity is centered near the maximal value of 1, which reveals that cHC droplets adopt a spherical shape. In contrast, the distribution of cHC-droplet sphericity is centered at 0.8, with a significant standard deviation of the sphericity distribution compared to the fHC droplets.

## IV. CONCLUSION

The spatial organization of the genome is not arbitrary and plays a pivotal role in gene regulation, replication, and repair. Chromosomes in interphase nuclei are organized into distinct territories (CTs), which are nonrandomly positioned with respect to the nuclear periphery and the nucleolus. Studies utilizing fluorescence *in situ* hybridization have delineated these territories, showcasing a radial pattern of genome organization [20,22]. The radial organization is rationalized by Hi-C analysis using graph theoretical techniques [21,52] and by polymer modeling of the *Drosophila* nucleus at resolution of individual topologically associating domains evolving throughout the interphase [19]. Eukaryotic nuclei, however, display a dynamic and active organization at all scales, throughout the interphase, senescence, diseases, and embryonic development [24,53-56]. Understanding the impact of various equilibrium and nonequilibrium physical forces and biochemical processes on shaping genome organization fuels one of the major unsolved challenges in biophysics, known as the four-dimensional nucleome project [57].

In the present paper we developed a multiphase liquid model of the nucleus, which can resolve chromosomal territories, compartments, and nuclear lamina in four dimensions using a physics-based and data-informed free-energy function. The MELON-4D model is a major improvement compared to the 2D mesoscale model we proposed previously [30]. MELON-4D enables rapid hypothesis-driven prototyping of 4D chromatin dynamics, facilitating learning of equilibrium and nonequilibrium driving forces from imaging experiments.

As an application of 4D modeling of eukaryotic nuclei, we explored the interplay of cohesive interactions in chromatin and the adhesive interaction of heterochromatin to the lamina, which influence the dynamics of the radial organization of heterochromatin. We mapped the phase diagram of nuclear morphologies of *Drosophila*, illuminating the interplay of forces favoring conventional, inverted, and senescent architectures. Interestingly, we found three distinct morphological phases with different heterochromatin connectivity patterns, which were observed in experiments on different eukaryotic nuclei at different developmental stages [24,58,59]. In agreement with the previous study of the *Drosophila* nucleus [19], we also found that the adhesive forces acting between heterochromatin and the lamina at the nuclear envelope are the dominant forces that disrupt chromatin radial organization. Disruption of radial order induced by reduced adhesive interactions leads to the variability of heterochromatin organization.

We have also explored the impact of heterochromatin-subtype interactions on the asymmetry of heterochromatin compartments. We have found that disparity in cohesive interactions between consecutive and facultative heterochromatin regulates connectivity and asymmetry of heterochromatin compartments without impacting the radial order. Finally, by employing the oblate shape of the nucleus, we quantified asymmetry in radial organization across major and minor axes of the nucleus, highlighting the importance of employing volumetric analysis of nuclear architecture.

A direct quantitative comparison between our simulations and experiments is not yet feasible due to a lack of experimental data and training sets for fitting parameters. However, the simulations of evolving chromatin patterns in three dimensions demonstrated the ability of the MELON-4D model to capture salient features of the chromatin architecture and dynamics. In the present study, we obtained sigmoidal shapes of heterochromatin radial profiles. Furthermore, MELON-4D revealed a phase diagram of nuclear architecture that captures different heterochromatin patterns seen in experiments, such as mesoscale channels and wetted droplet architectures.

We believe that mechanistic modeling of full nuclear dynamics in four dimensions will experience rapid growth in the future, and understanding the mechanobiology of the eukaryotic nucleus will take center stage in the quest to unravel the architecture-dynamics-function relation of the genome [10].
